# Rare Presentation of Imperforate Hymen in a 16‐Year‐Old: A Case Report From Nepal

**DOI:** 10.1002/ccr3.72482

**Published:** 2026-04-12

**Authors:** Dipendra Jung Shahi, Asmita Khanal, Nirmal Nagarkoti, Yamuna K.C, Santosh Upadhaya

**Affiliations:** ^1^ Department of Emergency Mugu District Hospital Mugu Karnali Province Nepal; ^2^ Department of Research Karnali Care International Hospital and Research Center Surkhet Karnali Province Nepal; ^3^ Department of Pediatrics Mugu District Hospital Mugu Karnali Province Nepal; ^4^ Department of Nursing Mugu District Hospital Mugu Karnali Province Nepal

## Abstract

Imperforate hymen should be suspected in adolescent girls presenting with primary amenorrhea and cyclic abdominal or pelvic pain. Early genital examination is essential for timely diagnosis. Prompt hymenectomy prevents hematocolpos, urinary obstruction, infection, and future reproductive morbidity.

## 1 Introduction

Hemocolpos refers to the accumulation of menstrual blood within the vagina and is most commonly caused by obstructive uterovaginal anomalies such as imperforate hymen [1, 2]. Imperforate hymen is the most frequent congenital cause of vaginal outflow obstruction, with an estimated incidence ranging from 0.05% to 0.1% [3, 4]. The condition is often asymptomatic until puberty, when retained menstrual blood may lead to hematocolpos, hematometra, lower abdominal pain, urinary retention, constipation, and rarely infection or endometriosis [5].

The hymen is typically removed as part of the selecting process. Hymenectomy or hymenotomy is the definitive and effective surgical management for imperforate hymen. Delayed diagnosis and intervention, however, may result in psychological distress and sociocultural challenges, particularly in low‐resource settings where access to gynecological care is limited [3, 6].

Due to low understanding, cultural norms, and limited access to gynecologic examinations, delayed detection is still widespread in many low‐resource countries, despite the need for early diagnosis. Teenagers sometimes wait until serious symptoms appear before visiting medical facilities, which can result in needless morbidity. In order to raise awareness among primary care physicians, decrease diagnostic delays, and emphasize the significance of early clinical suspicion in cases of primary amenorrhea and pelvic discomfort, it is crucial to report uncommon or unusual presentations of imperforate hymen [7].

## 2 Purpose Statement

This case report aims to highlight a rare presentation of imperforate hymen in an adolescent girl from Nepal and emphasizes the importance of early genital examination and timely surgical management to prevent complications.

## 3 Case Presentation

A 16‐year‐old girl from a rural village in Mugu District, Nepal, presented with a 6‐day history of severe, dull lower abdominal pain without radiation. There was no associated fever, nausea, vomiting, diarrhea, urinary symptoms, abnormal bowel habits, history of trauma, or prior gynecological procedures. She had never menstruated despite normal development of secondary sexual characteristics corresponding to Tanner stage III.

## 4 Physical Examination

On examination, the patient was well‐nourished, conscious, and hemodynamically stable. Abdominal examination revealed a palpable, non‐tender lower abdominal mass equivalent to a 16‐week gravid uterus, suggestive of a fluid‐filled structure. External genital examination demonstrated an imperforate hymen with a bulging membrane measuring approximately 4 × 4 cm, which became more prominent during the Valsalva maneuver. No discharge or other genital abnormalities were noted.

## 5 Differential Diagnosis

The differential diagnoses considered included transverse vaginal septum, vaginal agenesis, other Müllerian duct anomalies, and pelvic masses such as ovarian cysts.

## 6 Investigations

Pelvic ultrasonography revealed a markedly distended vaginal cavity filled with echogenic fluid consistent with retained menstrual blood (hematocolpos), confirming the diagnosis of imperforate hymen.

## 7 Management

The patient underwent hymenotomy under aseptic conditions, involving a cruciate incision of the hymenal membrane to allow drainage of accumulated menstrual blood. The procedure was completed without intraoperative complications.

## 8 Outcome and Follow‐Up

**FIGURE 1:**
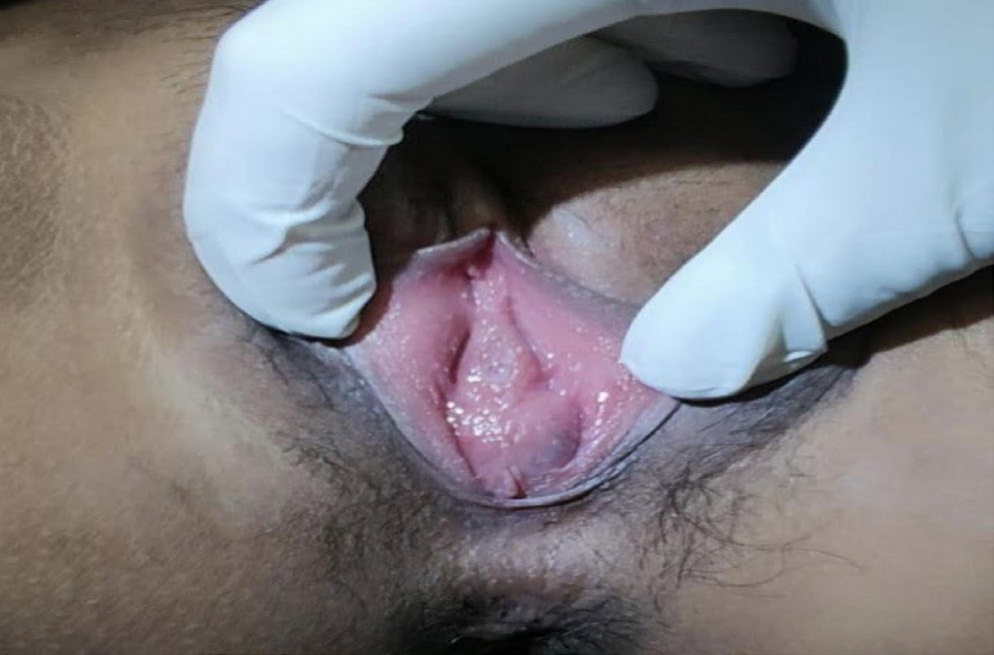
Imperforate before surgery.

**FIGURE 2:**
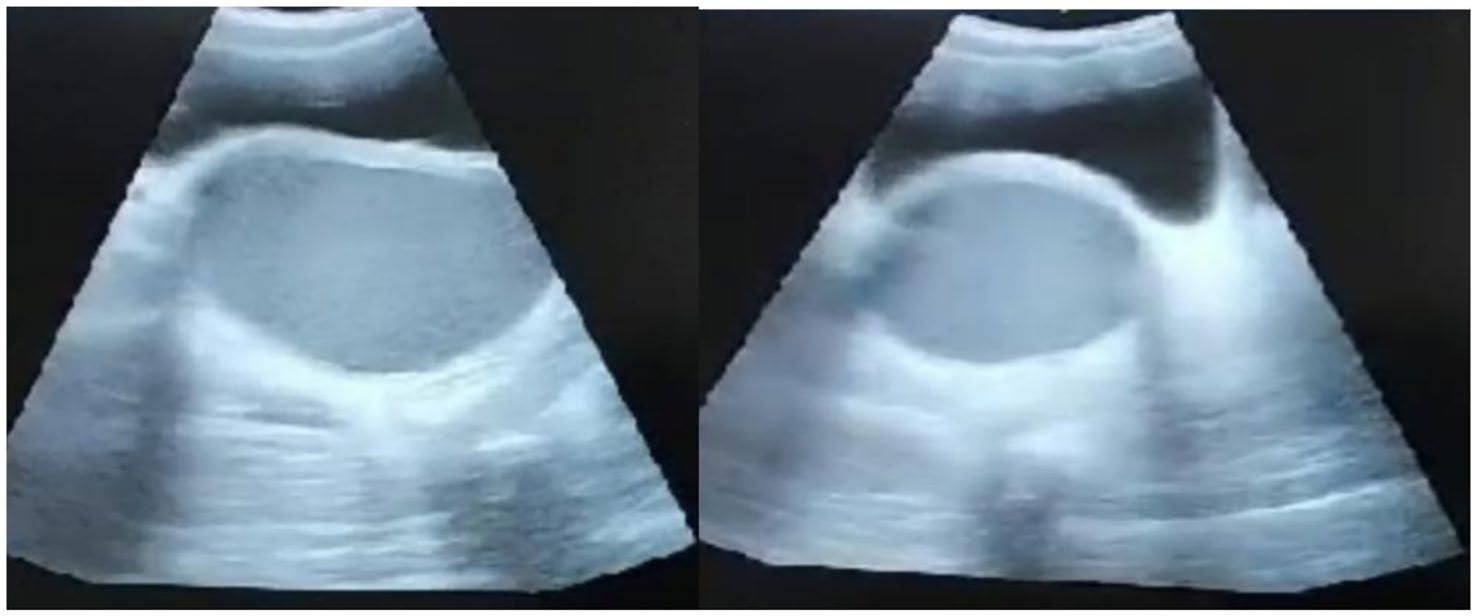
Presence of Hematocolpos (Ultrasound Scan).

**FIGURE 3:**
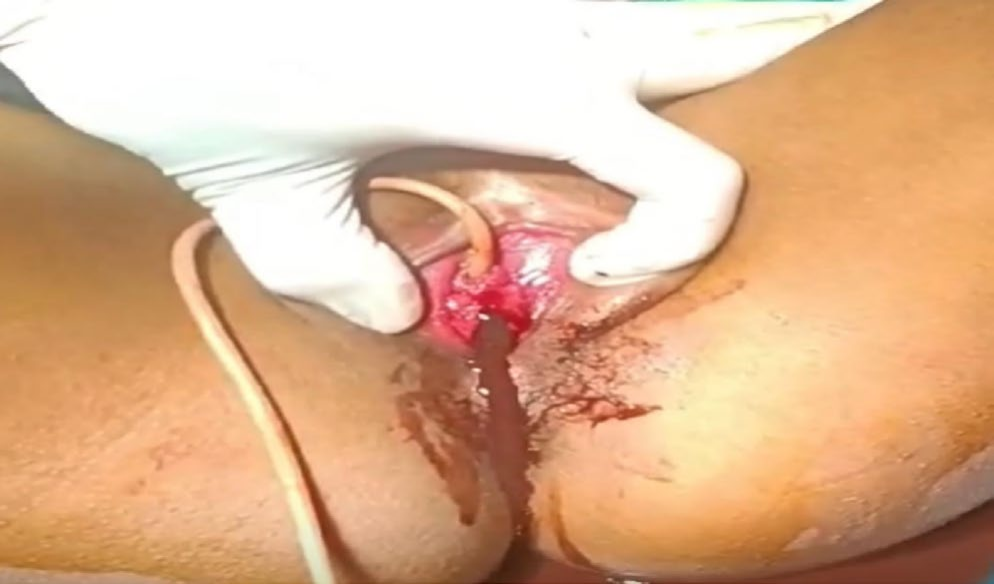
During Procedure.

**FIGURE 4:**
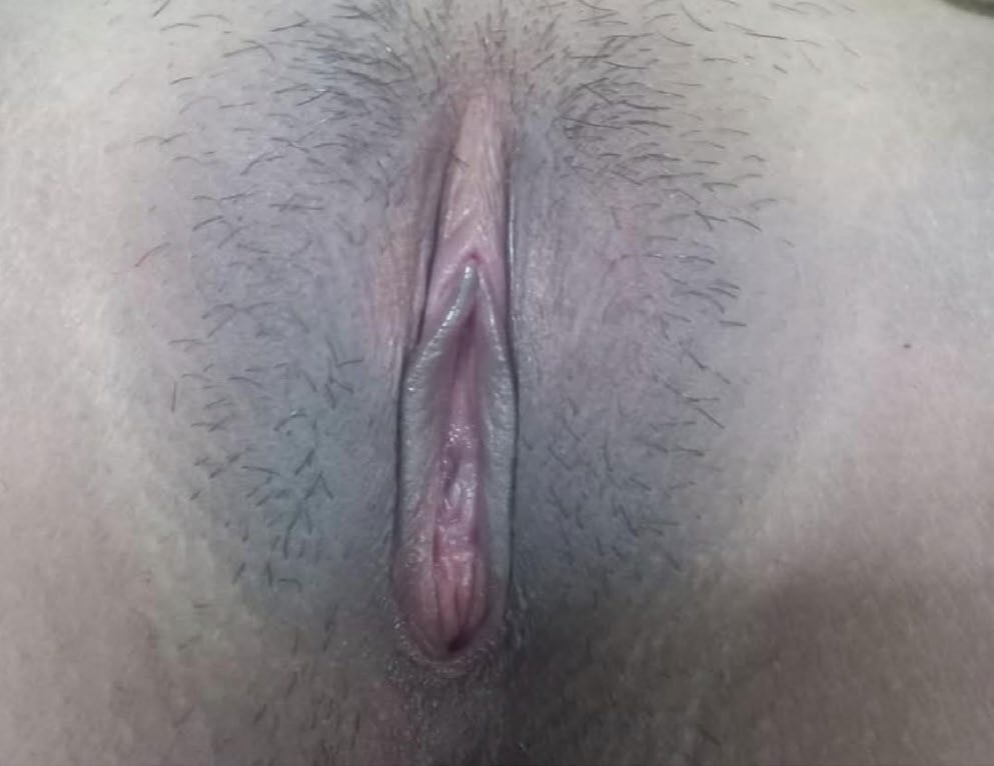
After Treatment.

The patient experienced marked clinical improvement following surgery, with complete resolution of abdominal pain. Follow‐up at 1 month and 7 months postoperatively confirmed regular menstrual cycles and absence of complications Figures 1, 2, 3, 4.

## 9 Conclusion

The patient's symptoms, including abdominal pain and primary amenorrhea, resolved following the hymenotomy. Post‐surgical follow‐up at one and 7 months confirmed the remission of symptoms, with regular menstrual periods and no problems. The timely surgical surgery avoided long‐term reproductive health complications.

This instance emphasizes the necessity of early detection and treatment, especially in remote regions, to avoid problems and improve reproductive health outcomes for adolescent girls.

## 10 Discussion

A rare congenital condition known as imperforate hymen is brought on by the hymenal membrane's incomplete degradation during fetal development. With an estimated incidence of 0.05%–0.1%, it is acknowledged as the most prevalent obstructive deformity of the female lower genital tract notwithstanding its rarity [3]. Due to the normal development of secondary sexual features, such as breast and pubic hair growth, in affected girls, the disorder typically goes undiagnosed until adolescence.

When menstruation begins, the blocked hymenal membrane stops menstrual blood from flowing out, which might result in hematosalpinx, hemocolpos, or hemometra. This causes symptoms including constipation, urine retention, ongoing lower abdominal pain, primary amenorrhea, and occasionally acute abdominal pain [5]. Due to societal hurdles, hesitation to perform genital examinations, and limited access to gynecologic care, the diagnosis may be delayed in many teenagers, particularly in low‐resource or remote places.

The patient in the present case had primary amenorrhea, a palpable lump, and lower abdominal pain, all of which are typical signs of hemocolpos. Significant accumulation of retained menstrual blood prior to presentation was indicated by the abdominal bulk similar to a 16‐week gravid uterus. This is consistent with studies from comparable rural settings, where late detection is caused by delays in seeking medical attention and a lack of diagnostic resources [8].

Early detection is critical since prolonged retention of menstrual blood can lead to issues such as endometriosis, pelvic adhesions, infection, infertility, and urinary tract blockage [9]. Ultrasonography is often sufficient to confirm the diagnosis, as it clearly demonstrates a fluid‐filled, distended vaginal and uterine cavity.

This case demonstrates why clinicians should examine imperforate hymen when an adolescent girl appears with primary amenorrhea and abdominal pain. Increasing awareness among primary care professionals, particularly in remote locations such as Mug, Nepal, can help to reduce diagnostic delays and prevent complications. In most situations, a basic clinical examination combined with an ultrasound is sufficient to make the diagnosis, allowing for timely surgery and a successful recovery.

## Author Contributions

**Dipendra Jung Shahi:** conceptualization, data curation, formal analysis, investigation, supervision, validation, visualization, writing – original draft. **Asmita Khanal:** conceptualization, data curation, formal analysis, investigation, methodology, project administration, software, supervision, validation, visualization, writing – original draft, writing – review and editing. **Nirmal Nagarkoti:** conceptualization, data curation, investigation, resources, validation, visualization, writing – review and editing. **Yamuna K.C:** data curation, investigation, supervision, visualization, writing – original draft. **Santosh Upadhaya:** conceptualization, data curation, investigation, visualization, writing – review and editing.

## Funding

The authors have nothing to report.

## Disclosure

No material from other sources was reproduced in this manuscript. All images and data presented are original and have been generated specifically for this case report. All authors certify that:

1. Each author meets the ICMJE criteria for authorship, including:
  ○ Substantial contribution to conception, design, acquisition, analysis, or interpretation
  ○ Drafting or critically revising the work for important intellectual content
  ○ Approval of the final version
  ○ Accountability for all aspects of the work
2. No person outside the listed authors made substantial contributions to the manuscript that would justify authorship.
3. No ghostwriter or third‐party agency has written or edited the manuscript in a manner that violates publication ethics. AI tools, if used, were for grammar assistance only and did not generate study content.
4. All data, images, and patient information used in the manuscript are original, and no plagiarism or duplication is involved.

## Ethics Statement

This case study was permitted by Mug District Hospital, Karnali Province Nepal. The study adhered to ethical guidelines to ensure patient confidentiality and informed consent.

## Consent

Written informed consent was obtained from the patient's legal guardian for the publication of this case report and associated clinical images. Identifying information has been removed to protect patient privacy.

## Conflicts of Interest

The authors declare no conflicts of interest.

## Data Availability

The datasets generated and analyzed during the current study are available from the corresponding author upon reasonable request.
